# A modified intrafocal pinning technique with three‐dimensional planning to facilitate volar plating in dorsally comminuted AO/OTA C2 and C3 distal radius fractures

**DOI:** 10.1186/s12891-021-04265-x

**Published:** 2021-04-23

**Authors:** Xue-yang Gui, Hong-fei Shi, Jin Xiong, Yi-xin Chen, Jun-fei Wang, Jie Huang, Xu-sheng Qiu, Yin-he Wang

**Affiliations:** Department of Orthopedics, Nanjing Drum Tower Hospital, The Affiliated Hospital of Nanjing University Medical School, No. 321 Zhongshan Road, Nanjing, China

**Keywords:** Distal radius fracture, Intrafocal pinning technique, Volar plating, Preoperative virtual planning

## Abstract

**Backgrounds:**

Theaim of this study was to assess the efficacy of a modified intrafocal pinningtechnique with three-dimensional (3D) planning to facilitate volar plating in dorsally comminuted intra-articular distal radius fractures.

**Methods:**

Intotal 35 AO/OTA type C2 and C3 fractures were finally included.The 3D digital model of the fracture was reconstructed based on preoperative computedtomographic (CT) images, with the displacement of the comminuted dorsalfragment and the intra-articular fragment analyzed for preoperative planning. During operation, amodified intrafocal pinning technique was applied percutaneously from thedorsal aspect of the radius to reduce the collapsed intra-articular fragmentfollowing volar plating. Adequate reduction was confirmed in all of patientsconsidering radial height, radial inclination and volar tilt in postoperativeradiographs.

**Results:**

No significant fracture re-displacement wasobserved in most of the cases during a mean follow-up period of 17.4 months, exceptfor two patients withthe C3 fracture. All of the patients achieved adequate clinicalROMs at 12 months postoperatively, with a mean DASH score of 12.0. Most of the patients achievedan excellent (*n* = 21) or good (*n* = 12) Gartland and Werley wrist score.

**Conclusions:**

Ourmodified intrafocal pinning technique with 3D planning contributes to a satisfactoryclinical and radiological outcome in dorsally comminuted intra-articular distalradius fractures fixed with a volar locking plate.

**Trialregistration:**

Notapplicable because the design of the study is retrospective.

## Introduction

Distal radius fracture is the second most common fracture in the elderly. Dorsal comminution occurs in about 60 % of the distal radius fractures, which leads to a reduced stability of fracture fixation, and an increased probability of fracture re-displacement [1]. Volar plating remains the most popular fixation techniques for the distal radial fractures because of the safe and straightforward surgical approach [2]. For dorsally comminuted distal radius fractures, especially those with dorsal articular compression, dorsal approach and plating were recommended to directly visualize the fracture fragment and to provide sufficient dorsal buttress [3]. However, a higher incidence of complications was reported to be associated with dorsal plating, which called for a less invasive method to facilitate fracture reduction and fixation for dorsally comminuted distal radius fractures [4–6].

The intrafocal pinning technique, described by Kapandji in 1987, was defined as the insertion of pins into the fracture site to lever the displaced fracture fragment into position [7]. Previously, the technique was commonly indicated for extra-articular fracture of distal radius with minimal dorsal and volar comminution [8]. Recent studies confirmed the feasibility of the intrafocal pinning technique as a reduction tool for unstable intra-articular fracture of distal radius fixed with volar locking plate [3, 9, 10]. In these studies, the dorsal cortex was considered crucial for the successful buttress effect provided by the intrafocal K-wires. The application of the intrafocal pinning technique in dorsally comminuted intra-articular distal radius fractures has never been investigated. In this study, we present a modified intrafocal pinning technique with three-dimensional (3D) planning to facilitate the reduction and fixation of dorsally comminuted intra-articular distal radius fractures.

## Methods

From January 2017 to Dec 2018, 41 consecutive patients with dorsally comminuted intraarticular distal radius fractures were enrolled according to the inclusion criteria of (1) age between 18 and 70 years, (2) AO/OTA type C2 and C3 fractures, (3) with dorsal metaphyseal comminution, and (4) meeting the indications for open reduction and internal fixation (ORIF) recommended on the American Association of Orthopaedic Surgeons (AAOS) standard [11]. Patients with delayed fractures, open fractures, neurovascular injuries, or additional ipsilateral upper extremity fractures were excluded. The study protocol was approved by our institution’s Medical Ethics Committee and the informed consent was obtained.

**Fig. 1:**
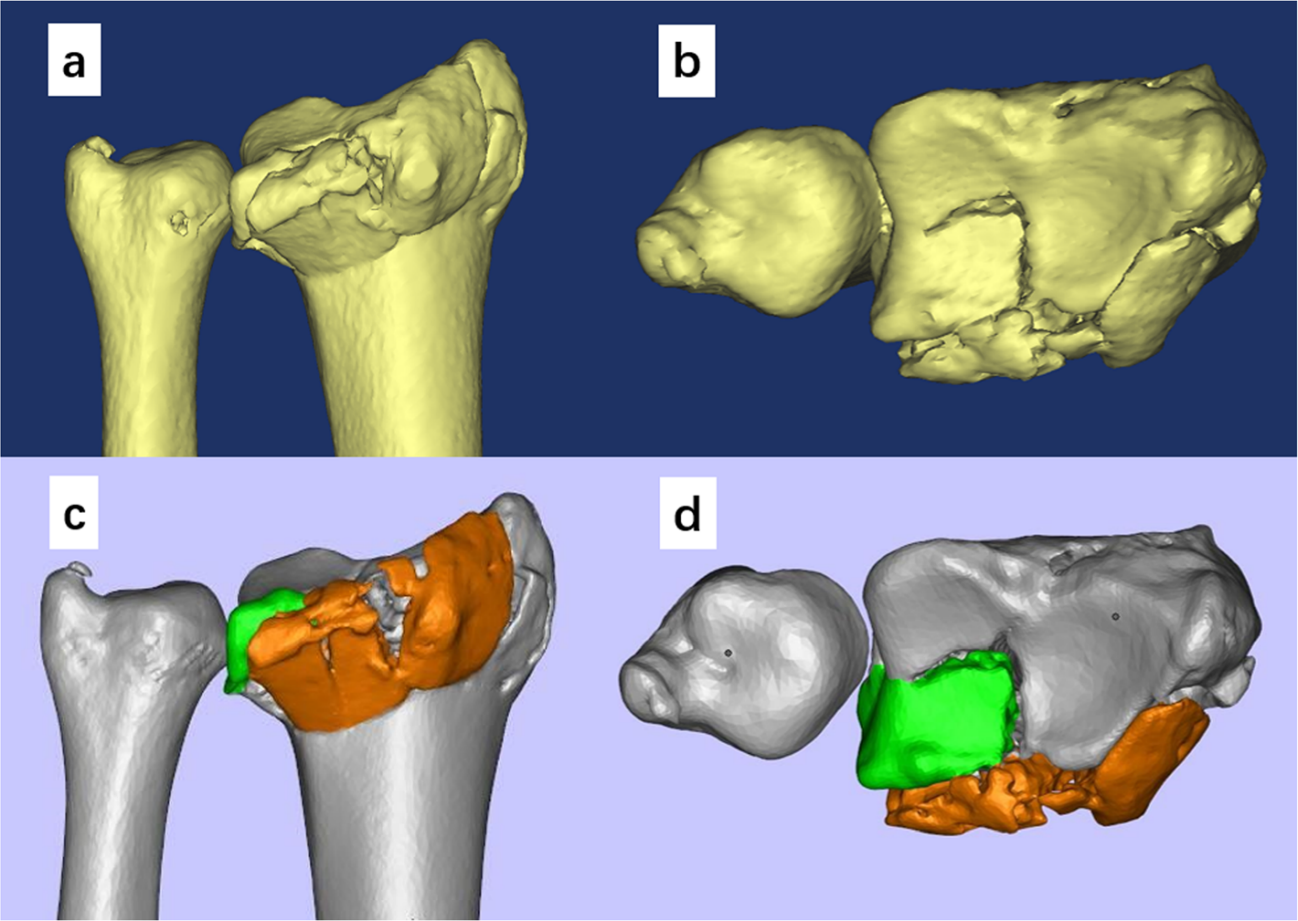
The 3D digital model was reconstructed to analyze the feature of the fracture. The dorsal view (**a**) and the top view (**b**) of the distal radius were shown in Mimics software to identify the displacement of the dorsal fragment and the articular step. In Magics software, the dorsal fragment (orange) and the intra-articular fragment (green) were identified and assigned different colors (**c** and **d**)

**Fig. 2:**
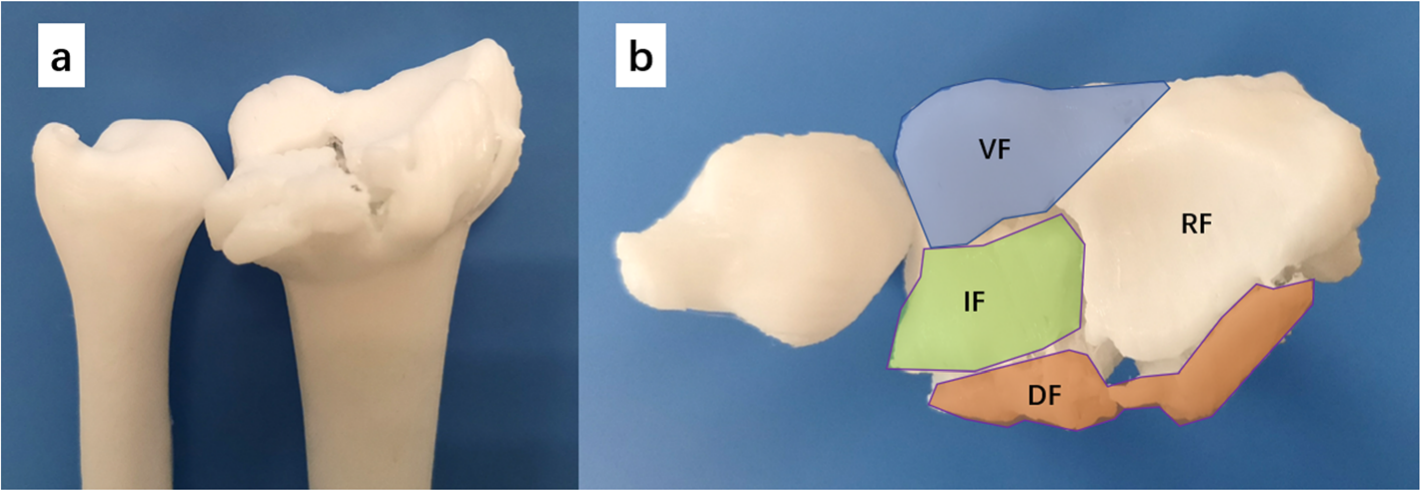
The dorsal view (**a**) and the top view (**b**) of the 3D printed model was shown to evaluation of the features of the fracture. VF = volar fragment; IF = intra-articular fragment; RF = radial column fragment; DF = dorsal fragment

Preoperative posteroanterior (PA) and lateral radiographs, as well as computed tomographic (CT) images were evaluated to classify the fractures according to AO/OTA system. CT scan (GE Revolution CT, America) were performed using the following parameters: 130 kV, 21.6mAs, and 0.625 mm layer thickness. The CT images were exported from the Picture Archiving and Communication System (PACS) as Digital Imaging and Communications in Medicine (DICOM) files and imported to the Mimics 15.0 software (Materialise, Leuven, Belgium). The bony structure around the wrist joint was revealed by adjusting the threshold. The fractured distal radius was separated by applying the Region Growing function during segmentation. The 3D digital model of the fracture was then reconstructed and analyzed (Fig. 1a and b). The displacement of the dorsal fragment and the articular incongruency (step or gap) were identified and assigned different colors in Magics 21.0 software (Materialise, Leuven, Belgium) (Fig. 1c and d). The 3D model exported as a binary stereolithography (STL) file was then sent to the 3D printer (Flashforge Guider 2 S, USA) for 3D printing. The 3D printed model was used to simulate the fracture reduction and to provide guidance for the entry point and orientation of intrafocal pinning (Fig. 2).

**Fig. 3:**
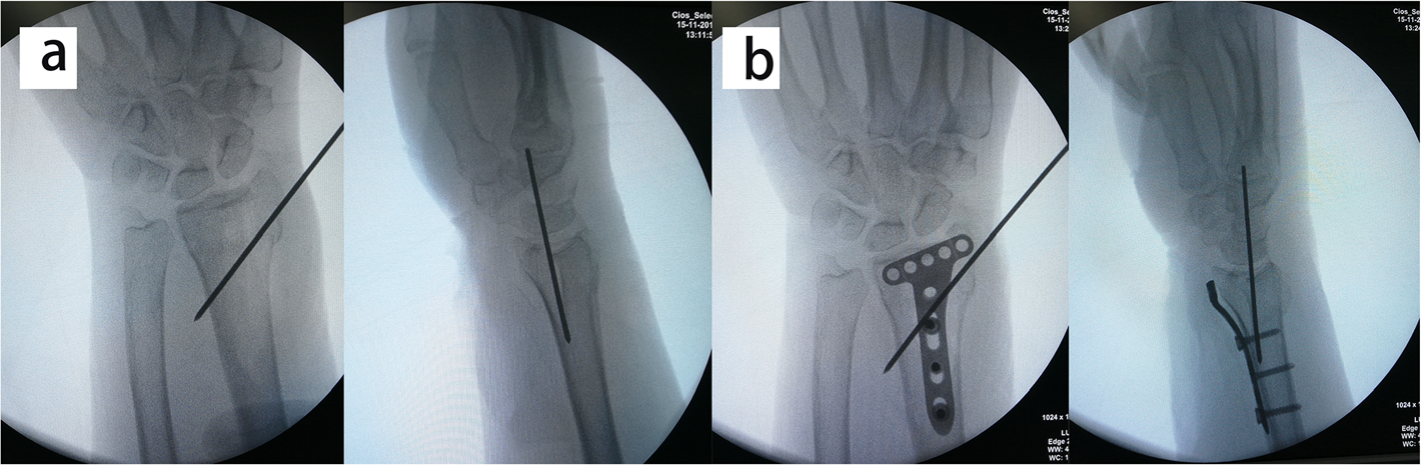
The intraoperative C-arm images showing the temporal fixation of the volar fracture fragment. **a** Following longitudinal traction, the volar fragments were reduced and temporarily fixed with K-wires; **b** A 2.4-mm volar distal radial locking plate was applied with the proximal end of the plate fixed to the proximal radial fragment

**Fig. 4:**
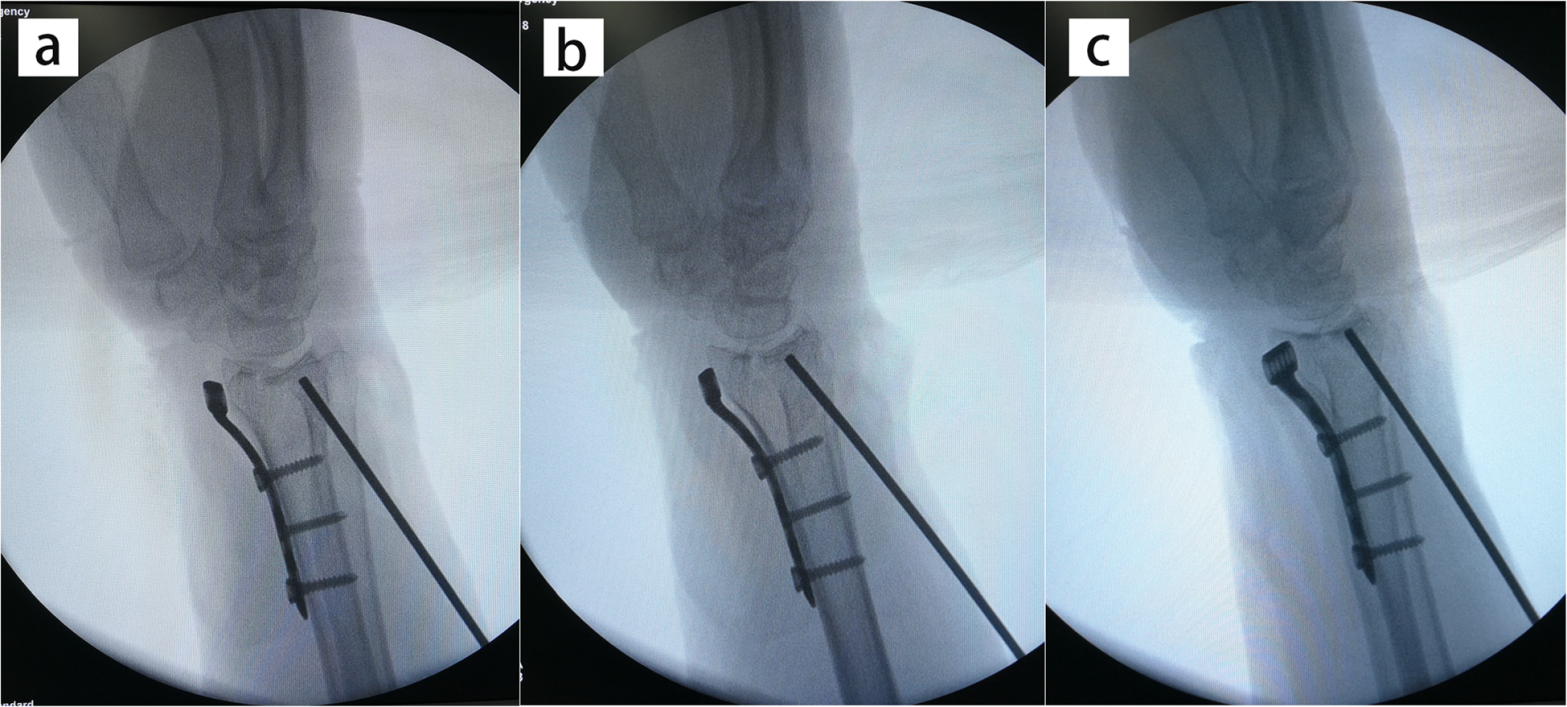
A series of the intraoperative C-arm images showing the insertion of the K-wire through the fractured dorsal cortex (**a**) to elevate intra-articular impaction (**b** and **c**)

**Fig. 5:**
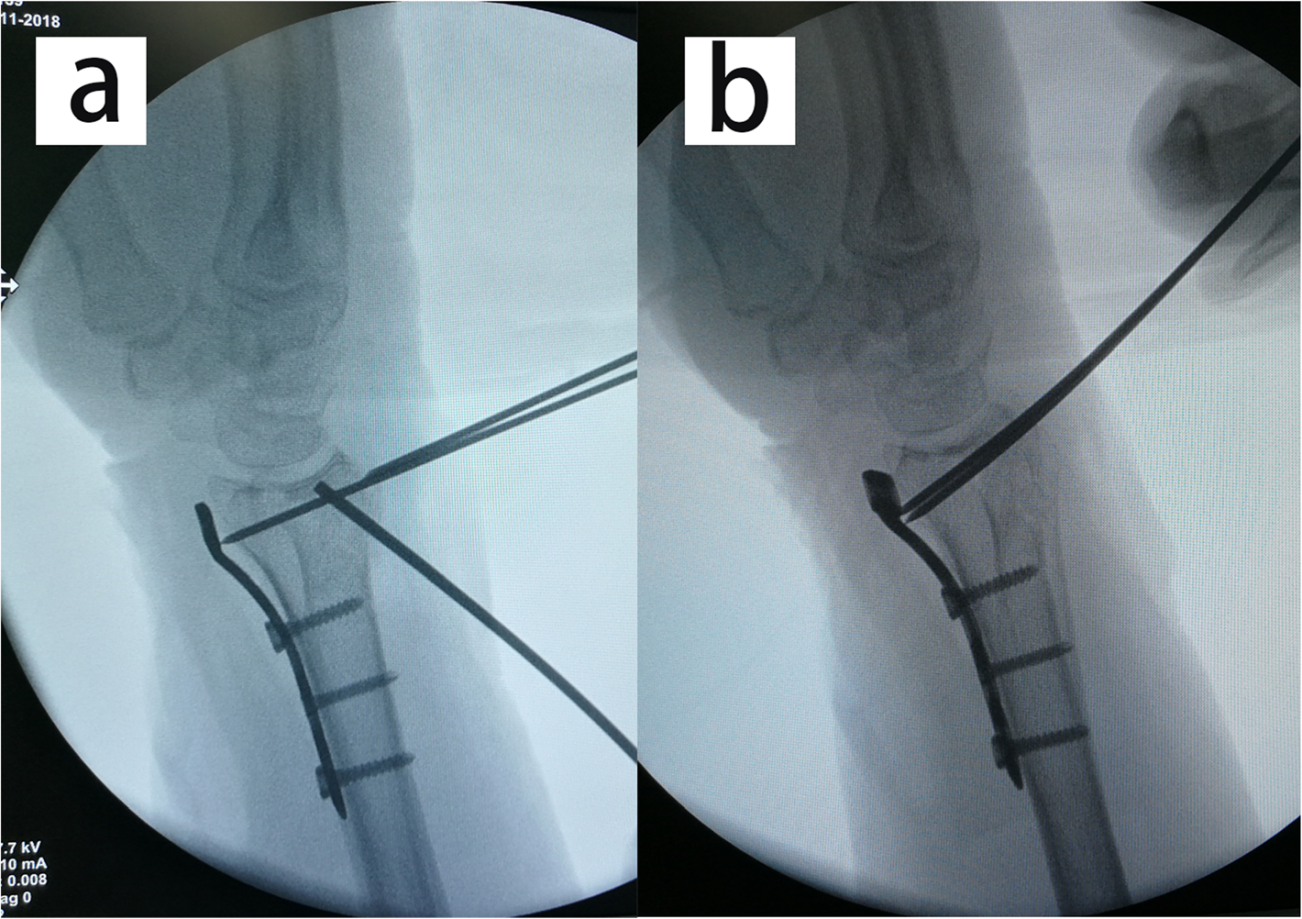
A series of the intraoperative C-arm images showing the levering maneuver using multiple subchondral pins to recover the volar tilt

**Fig. 6:**
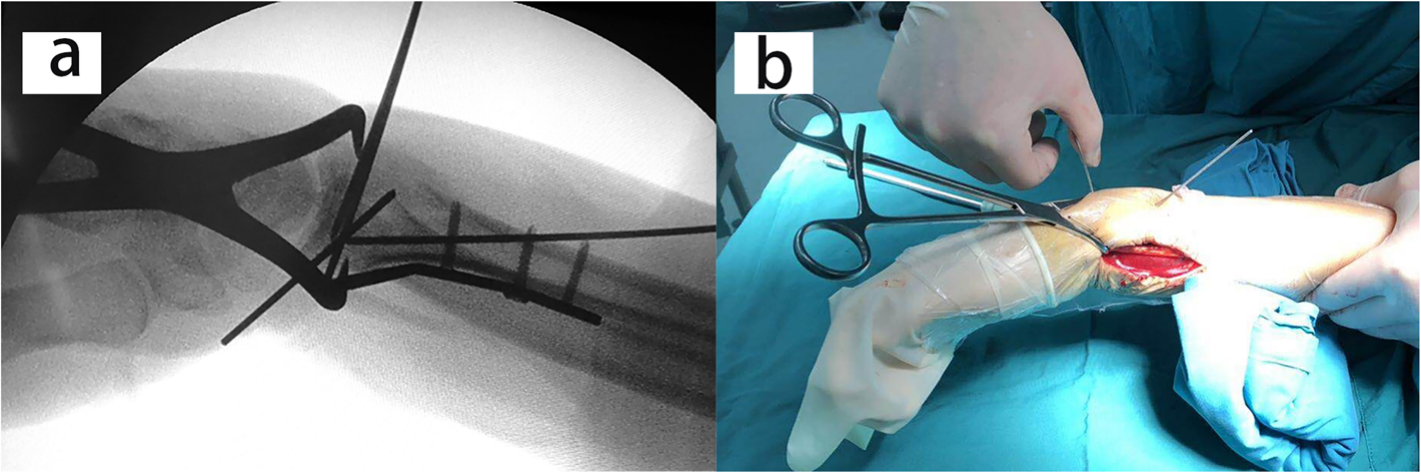
Percutaneous clamping technique to reduce the fracture gap in coronal plane. **a** The C-arm images showing percutaneous clamping; **b** Percutaneous clamping and the levering maneuver described in Fig. 5

**Fig. 7:**
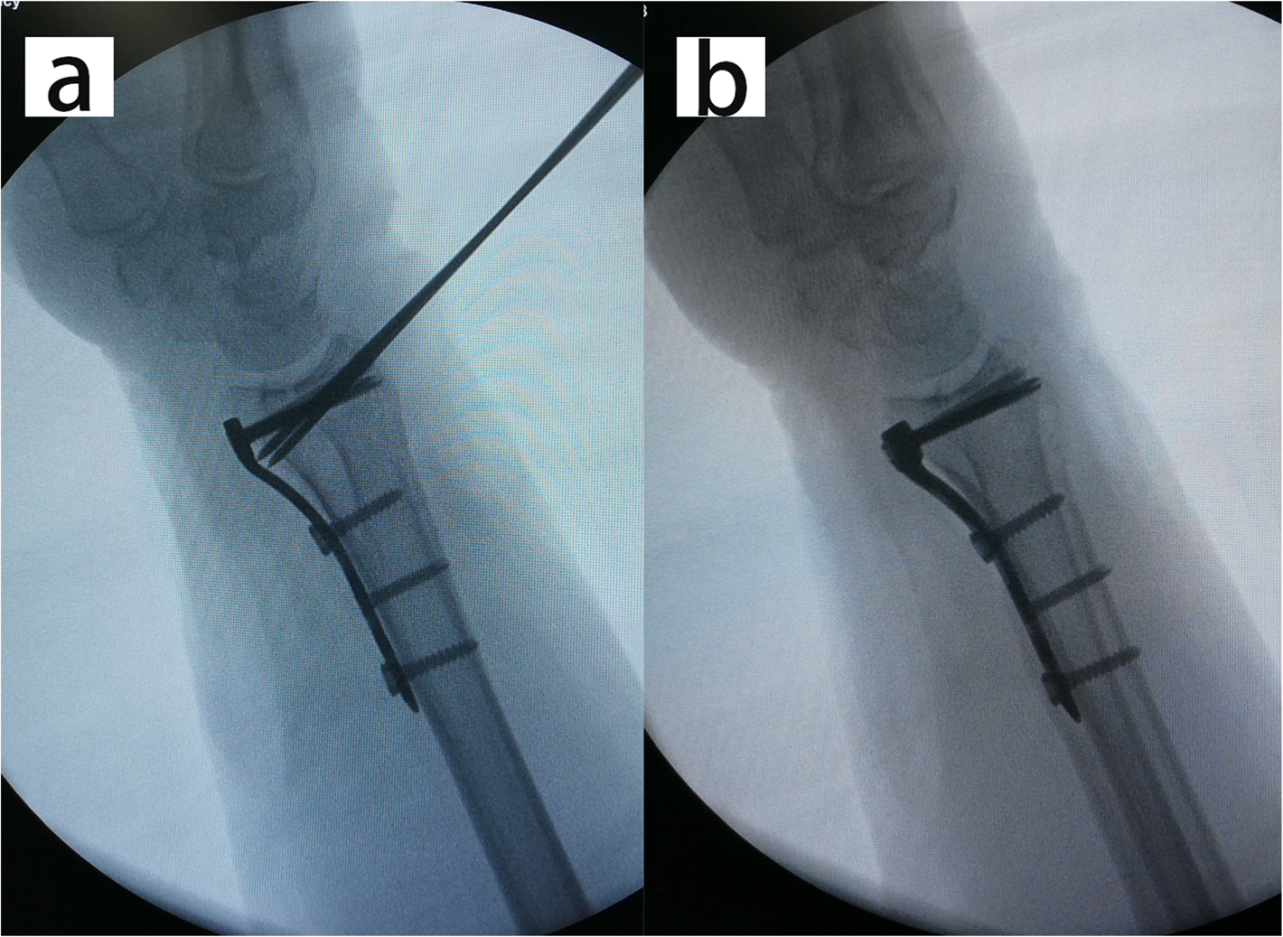
A series of the intraoperative C-arm images showing the fixation of intra-articular fragment and the dorsal fragment with distal locking screws inserted through the volar plate

The operation was performed within 2 weeks after injury. All patients were operated on by one senior attending surgeon. During operation, the arm was placed on a radiolucent side table with a tourniquet applied to the upper arm. The modified volar Henry approach was used with the longitudinal skin incision performed in line with the flexor carpi radialis (FCR) tendon. With the FCR tendon and the flexor pollicis longus (FPL) retracted ulnarly, the pronator quadratus was exposed and released from the radius with an inverted L-shaped incision. With longitudinal traction maneuver, the fracture was provisionally reduced. According to the integrity of the metaphyseal cortex, either the radial column or the palmoulnar fragment could be reduced first to provide reference for the radial height and radial inclination. The volar fragments were then reduced and temporarily fixed with K-wires. A 2.4-mm volar distal radial locking plate (Depuy-Synthes, Oberdorf, Switzerland) was applied with the proximal end of the plate fixed to the proximal radial fragment (Fig. 3). Intrafocal pinning technique was applied percutaneously from the dorsal aspect of the radius based on preoperative virtual planning and the printed model. Namely, a K-wire (1.5 mm or 2 mm diameter) was inserted through the fractured dorsal cortex to reduce the collapsed intra-articular fragment under fluoroscopic guidance (Fig. 4). The fracture gap in the coronal plane could be reduced using percutaneously applied pointed reduction forceps. Multiple subchondral pins were then inserted percutaneously from the dorsal aspect to fix the reduced intra-articular fragment and the dorsal fragment. To restore the volar tilt, the subchondral pins could be used to lever the distal fragment (Figs. 5 and 6). The distal locking screws were then inserted through the volar plate to secure the intra-articular and dorsal fragment (Fig. 7). Reduction and fixation were verified using intraoperative C-arm. The stability of the distal radioulnar joint (DRUJ) was routinely checked and compared with the contralateral side. Cast immobilization, radioulnar pinning, or ulnar styloid ORIF was performed based on the instability of DRUJ according to the established protocol [12, 13].

Postoperative radiographs and CT were routinely checked to evaluate the quality of fracture reduction and fixation. Clinical and radiological assessments were performed at 6 weeks, 3 months, 6 months, and 12 months postoperatively according to our routine follow-up regime [12]. Radiological parameters including radial inclination, volar tilt, and radial height were measured on the PACS according to the protocols described in literature [14]. Differences in the radiological parameters between the postoperative values and those taken at the 12-month follow-up were compared using paired-samples t test. Clinical assessment and complications of included patients were recorded during follow-up. Patient wrist range of motion (ROMs), pain, and functional outcomes were evaluated according to the Disabilities of the Arm, Shoulder and Hand (DASH) score and the Gartland and Werley score at 12 months postoperatively. For the statistical tests, *P* < 0.05 was considered statistically significant. Statistical Product and Service Solutions (SPSS) software (SPSS version 18.0, SPSS, IBM Inc., Armonk, NY, USA) was used for all statistical analyses.

## Results

Among the 41 patients enrolled during the study period, 35 were eligible for the final analysis. The other 6 patients were excluded because of a follow-up period less than 12 months. Patients’ demographics were shown in Table 1. There were 21 AO/OTA type C2 and 14 AO/OTA type C3 fractures. The mean age was 62.2 (29 to 83) years. The average follow-up time was 17.4 (12–24) months.

**Table 1:** Demographic data of the patients

	Number
Age (years)	
Mean (min, max)	62.2 (29 to 83)
Gender	
Male	12 (34.28 %)
Female	23 (65.71 %)
AO/OTA classification	
C2	21 (60 %)
C3	14 (40 %)
follow-up time (months)	
Mean (min, max)	17.4 (12 to 24)

Bone healing was achieved in all patients during follow-up. The union of bone was observed at 6 weeks postoperatively in 27 patients, and at 3 months in the other 8 patients. One patient had a superficial wound infection postoperatively. The incision healed smoothly after dressing changes and antibiotic treatment. No serious complication, such as nerve injury, tendon rupture, or implant failure was recorded during follow-up.

**Fig. 8:**
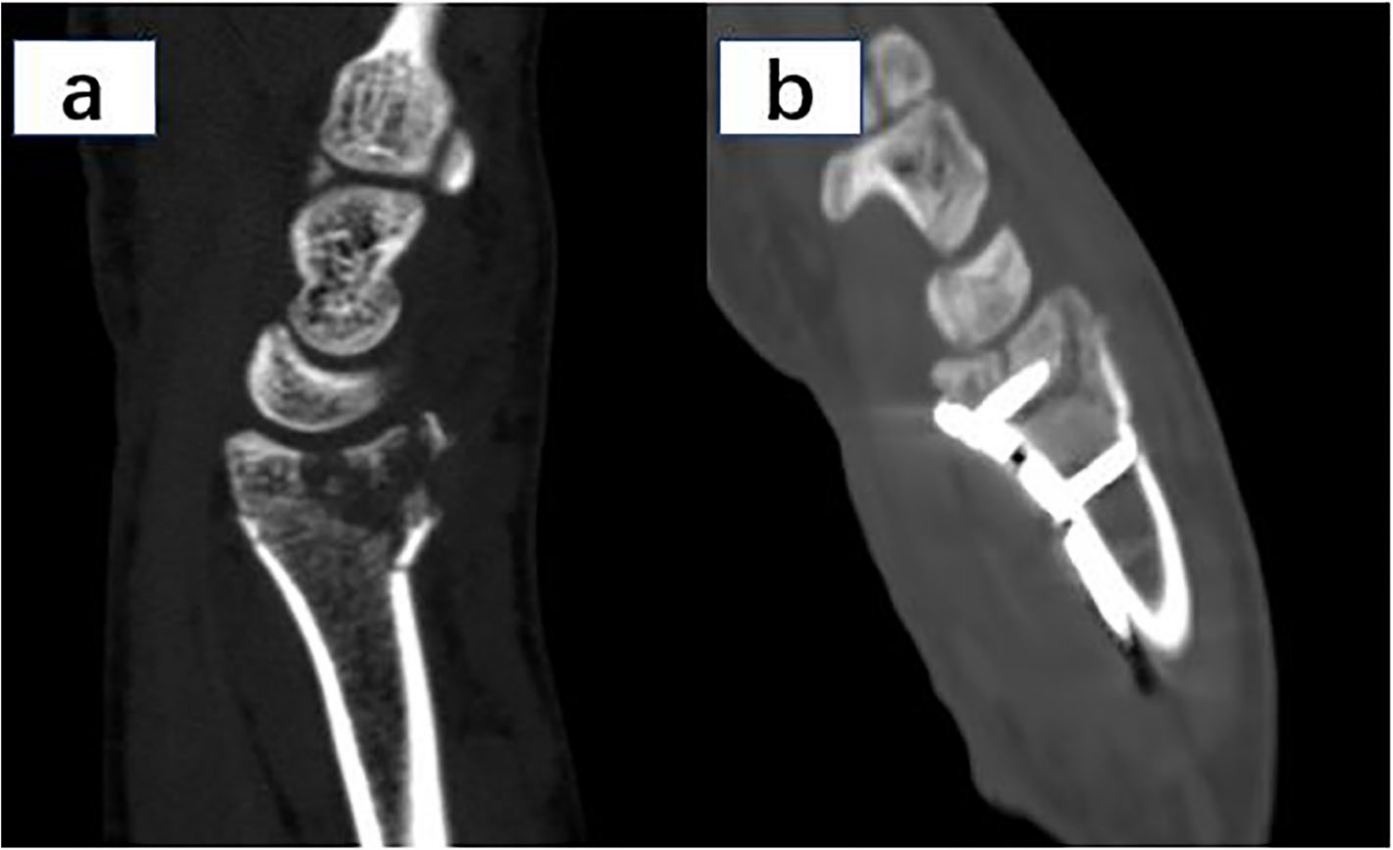
Pre- and post-operative sagittal view CT images showing the reduction and fixation of the articular fragment. **a** Pre-operative CT image showed the articular step and gap, as well as the dorsal comminution of the fractured distal radius; **b** Post-operative CT image showed the articular step and gap were well reduced

Adequate reduction was achieved in all patients considering radial height, radial inclination and volar tilt immediately postoperatively (Fig. 8). The immediate postoperative radial height was 10.4 ± 3.3 mm, the radial inclination was 20.6 ± 4.2 degrees, and volar tilt was 6.6 ± 4.6 degrees. Comparing the final follow-up radiographs with those taken immediate postoperatively, no statistically-significant change in volar tilt (*P =* 0.539), radial inclination (*P =* 0.142) or radial height (*P =* 0.275) was observed (Table 2). Most of the patients maintained good reduction during follow-up, except for two patients with C3 fracture who presented significant re-displacement of the dorsal fragment with the change of the volar tilt measured to be 12°and 15°respectively.

**Table 2:** Radiographic evaluation during follow-up (Mean ± SD)

Variables	Postoperative	Final follow-up	P value*
Radial inclination (°)	20.6±4.2	20.2±3.7	0.142
Volar tilt (°)	6.6±4.6	6.2±4.4	0.539
Radial height (mm)	10.4±3.3	10.2±3.5	0.275

*Comparison of Postoperative and Final follow-up parameters. Paired *t* test

At 12 months postoperatively the mean ROMs in the injured side were measured to be: extension 71.3 ± 6.7 degrees, flexion 70.2 ± 4.6 degrees, pronation 82.4 ± 4.9 degrees, and supination 85.7 ± 5.1 degrees. No significant difference was observed compared with the contralateral normal wrist in pronation and supination, while statistically significant differences were observed in extension and flexion (Table 3). All of the patients achieved adequate clinical ROMs according to Ryu’s standard [15].

**Table 3:** Comparison of the range of motion between the Injured and the Contralateral normal wrist at 12 months postoperatively (Mean ± SD)

Variables	Injured Side	Contralateral Side	P value*
Extension (°)	71.3±6.7	82.6±7.2	0.016
Flexion (°)	70.2±4.6	87.1±5.4	<0.01
Pronation (°)	82.4±4.9	82.9±4.5	0.520
Supination (°)	85.7±5.1	86.1±5.5	0.383

*Paired *t* test

**Fig. 9:**
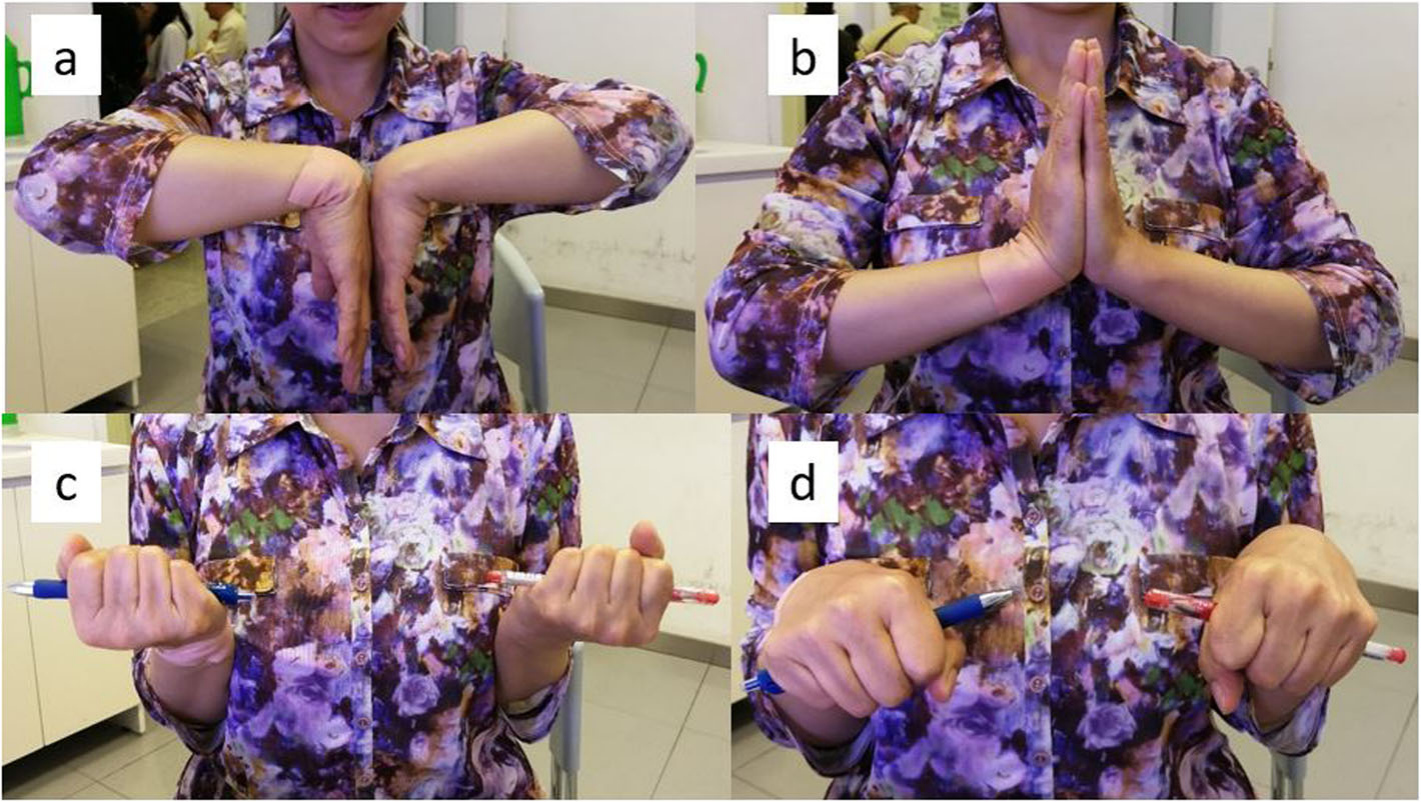
The Range of motion of a 55 years old female patient with AO23-C2 fracture on her right extremity. The flexion (**a**), extension (**b**), supination (**c**) and pronation (**d**) of the wrist recovered well in 12 months after surgery

The mean DASH score was 12.0 (0–57) at 12 months follow-up. Considering different fracture types, the DASH scores were 10.2 and 13.4 in AO/OTA C2 and C3 patients respectively. Most of the patients achieved an excellent (*n* = 21) or good (*n* = 12) Gartland and Werley wrist score (Fig. 9), except for the two patients presented significant loss of volar tilt who resulted in a fair functional outcome.

## Discussion

In this study, a modified intrafocal pinning technique with 3D planning was used to facilitate the reduction and fixation of dorsally comminuted intra-articular distal radius fractures following volar plating. Postoperative radiographic and clinical assessment showed a satisfactory reduction and good to excellent outcomes in most of the patients.

The original intrafocal pinning technique described by Kapandji was used with the pins maintained throughout the fracture healing period [16]. This technique was proven to provide sufficient buttress against dorsal extra-articular fractures of distal radius [16]. On the other hand, the limitations of this pinning technique did exist. Some studies found that elderly patients with osteoporosis did not achieve good functional outcome using the original intrafocal pinning technique [8, 17]. Besides, this technique was not recommended for intra-articular distal radius fracture with dorsal or volar comminution [16]. The integrity of the dorsal and volar metaphyseal cortex was considered crucial. Compared with the original Kapandji technique, we used intrafocal pins through the fractured dorsal cortex to reduce the collapsed intra-articular fragment. The prepositioned volar locking plate served as a buttress during this procedure, which eliminated the need for a relatively intact volar metaphyseal cortex. Besides, in our technique, multiple percutaneous subchondral pins from the dorsal aspect were applied to lever the articular surface and hence to restore the volar tilt. An extra dorsal approach or dorsal plating was not required in this case series.

Different modifications to the original Kapandji technique were reported in recent studies [3, 9, 10]. Huang et al. reported a modified sandwich method, in which the intrafocal K-wires were inserted from the dorsal and radial site to reduce the dorsal and radial fracture displacement. The anatomic palmar plate was then applied to build a sandwich structure for fracture fixation. The results showed that the sandwich method could achieve similar radial height, radial inclination, volar tilt, and ulnar variance comparing with the contralateral non-injured side [9]. Compared with our study, either the reduction of intra-articular depression or the situation of dorsal comminution was not specified in Huang’s report. Jirangkul et al. also reported the application of the additional volar locking plate following Kapandji intrafocal K-wire pinning [3]. A total of 57 cases of intra-articular fractures of the distal radius were treated, and most of cases achieved good to excellent clinical outcomes [3]. Compared with our study, the intra-articular fracture fragment was not reduced using intrafocal pinning technique in Jirangkul’s study. Besides, the situation of dorsal comminution was not addressed in this study.

In our study, the preoperative surgical planning was performed using 3D digital model in our technique to analyze the collapsed intra-articular fragment as well as the characteristics of the comminuted dorsal cortex. The feasibility of CT-based virtual preoperative planning has been investigated in previous studies [18–21]. Compared with the traditional preoperative planning techniques using handwriting hardcopy radiographs along with tracing paper or simple measurements in the PACS, the CT-based 3D preoperative planning was proven to provide better understanding of fracture shape and displacement, especially in intra-articular fractures [22, 23]. In our case series, the collapsed intra-articular fragment as well as the characteristics of the comminuted dorsal cortex were carefully analyzed preoperatively in the 3D digital model. Then, the 3D printed model was used to simulate the fracture reduction. The entry point and orientation of each specific pin were designed on the printed model, which improved the surgeon’s understanding of the fracture characteristics and helped the surgeon to be familiar with the reduction procedure.

Most of the patients (94.3 %) achieved excellent or good functional recovery in our study, probably due to the adequate fracture reduction including the recovery of radial inclination, volar tilt, and radial height, which were proven crucial for a satisfactory outcome [24, 25]. Considering different fracture types, all of the AO type C2 fractures resulted in good or excellent functional outcome. Compared with Chou’s study, in which an excellent or good outcome was achieved in 86.4 % (19/22) of the patients with C3 dorsally-comminuted distal radial fractures treated with dorsal plating, our study showed similar results in C3 fractures with 85.7 % (12/14) of the patients resulted in good or excellent functional recovery [26]. This indicated that our modified intrafocal pinning technique could facilitate volar plating and lead to similar clinical outcome as dorsal plating, even in dorsally comminuted C3 distal radius fractures.

This study had several limitations. First, the study included limited numbers of patients. More data might be needed to draw a solid conclusion on the treatment of these complex fractures. Besides, all the operations were performed within one institution by a single surgeon. Nevertheless, the bias owing to different surgeons’ experience and preference was prevented. Another limitation was the lack of comparison with other reduction techniques. Future studies with a larger sample size or with other technique as control group are needed to evaluate the clinical results.

## Conclusions

The modified intrafocal pinning technique with preoperative 3D planning in this study contributes to a satisfactory clinical and radiological outcome in dorsally comminuted intra-articular distal radius fractures with volar locking plate fixation.

## Data Availability

The datasets generated and/or analysed during the current study are not publicly available due to the regulations of IRB, but can be made available from the corresponding author on reasonable request.

## Abbreviations

3D: Three dimensional
CT: Computed Tomography
ORIF: Open reduction and internal fixation
AAOS: The American Association of Orthopaedic Surgeons
PA: Posteroanterior
PACS: Picture Archiving and Communication System
DICOM: Digital Imaging and Communications in Medicine
STL: Stereolithography
FCR: Flexor carpi radialis
FPL: Flexor pollicis longus
DRUJ: Distal radioulnar joint
ROM: Range of motion
DASH: Disabilities of the Arm, Shoulder and Hand score
SPSS: Statistical Product and Service Solutions
VF: Volar fragment
IF: Intra-articular fragment
RF: Radial column fragment
DF: Dorsal fragment

## References

[1] Makhni EC, Taghinia A, Ewald T, Zurakowski D, Day CS (2010). Comminution of the dorsal metaphysis and its effects on the radiographic outcomes of distal radius fractures. J Hand Surg Eur.

[2] Asadollahi S, Keith PP (2013). Flexor tendon injuries following plate fixation of distal radius fractures: a systematic review of the literature. J Orthop Traumatol.

[3] Jirangkul P, Jitprapaikulsarn S, Songpatanaslip T (2019). Outcomes Following Temporary Kapandji Pinning Technique and Distal Radial LCP Fixation for Intra-Articular Fractures of the Displaced Distal Radius. Tech Hand Up Extrem Surg.

[4] Farhan MF, Wong JH, Sreedharan S, Yong FC, Teoh LC (2015). Combined volar and dorsal plating for complex comminuted distal radial fractures. J Orthop Surg.

[5] Day CS, Kamath AF, Makhni E, Jean-Gilles J, Zurakowski D (2008). "Sandwich” plating for intra-articular distal radius fractures with volar and dorsal metaphyseal comminution. Hand (New York, NY).

[6] Alluri RK, Hill JR, Ghiassi A (2016). Distal Radius Fractures: Approaches, Indications, and Techniques. J Hand Surg.

[7] Kapandji A: [Intra-focal pinning of fractures of the distal end of the radius 10 years later]. An Chir Main 1987, 6(1):57–63. 10.1016/s0753-9053(87)80011-x 3619531

[8] Trumble TE, Wagner W, Hanel DP, Vedder NB, Gilbert M (1998). Intrafocal (Kapandji) pinning of distal radius fractures with and without external fixation. J Hand Surg.

[9] Huang HK, Huang YC, Wang JP, Chang MC (2017). A Sandwich Method Using Kapandji Intrafocal Pinning to Facilitate Palmar Plating of Displaced Distal Radius Fractures. Tech Hand Up Extrem Surg.

[10] Rubin G, Chezar A, Rinott M, Bor N, Rozen N (2013). Treatment of intra-articular distal radius fractures by the volar intrafocal Kapandji method: a case series. Tech Hand Up Extrem Surg.

[11] Lichtman DM, Bindra RR, Boyer MI, Putnam MD, Ring D, Slutsky DJ, Taras JS, Watters WC, Goldberg MJ, Keith M (2010). Treatment of distal radius fractures. J Am Acad Orthop Surg.

[12] Chen YX, Zheng X, Shi HF, Wangyang YF, Yuan H, Xie XX, Li DY, Wang CJ, Qiu XS (2013). Will the untreated ulnar styloid fracture influence the outcome of unstable distal radial fracture treated with external fixation when the distal radioulnar joint is stable. BMC Musculoskelet Disord.

[13] Sammer DM, Chung KC (2012). Management of the distal radioulnar joint and ulnar styloid fracture. Hand Clin.

[14] Kreder HJ, Hanel DP, McKee M, Jupiter J, McGillivary G, Swiontkowski MF (1996). X-ray film measurements for healed distal radius fractures. J Hand Surg.

[15] Ryu JY, Cooney WP, Askew LJ, An KN, Chao EY (1991). Functional ranges of motion of the wrist joint. J Hand Surg.

[16] Weil WM, Trumble TE (2005). Treatment of distal radius fractures with intrafocal (kapandji) pinning and supplemental skeletal stabilization. Hand Clin.

[17] Brady O, Rice J, Nicholson P, Kelly E, O’Rourke SK (1999). The unstable distal radial fracture one year post Kapandji intrafocal pinning. Injury.

[18] de Muinck Keizer RJO, Lechner KM, Mulders MAM, Schep NWL, Eygendaal D, Goslings JC (2017). Three-dimensional virtual planning of corrective osteotomies of distal radius malunions: a systematic review and meta-analysis. Strategies Trauma Limb Reconstr.

[19] Ma B, Kunz M, Gammon B, Ellis RE, Pichora DR (2014). A laboratory comparison of computer navigation and individualized guides for distal radius osteotomy. Int J Comp Assist Radiol Surg.

[20] Bizzotto N, Tami I, Tami A, Spiegel A, Romani D, Corain M, Adani R, Magnan B (2016). 3D Printed models of distal radius fractures. Injury.

[21] Xu J, Zhang G, He Z, Zhong S, Chen Y, Wei C, Zheng Y, Lin H, Li W, Huang W: Anatomical reduction and precise internal fixation of intra-articular fractures of the distal radius with virtual X-ray and 3D printing. Australas Phys Eng Sci Med. 2019;43(1):35–47. 10.1007/s13246-019-00795-w PMC7026237 31641940

[22] Bizzotto N, Tami I, Tami A, Spiegel A, Romani D, Corain M, Adani R, Magnan B (2016). 3D printed models of distal radius fractures. J Injury.

[23] Yoshii Y, Totoki Y, Sashida S, Sakai S, Ishii T (2019). Utility of an image fusion system for 3D preoperative planning and fluoroscopy in the osteosynthesis of distal radius fractures. J Orthop Surg Res.

[24] Porter M, Stockley I: Fractures of the distal radius. Intermediate and end results in relation to radiologic parameters. Clin Orthop Relat Res 1987(220):241–252. 3594997

[25] Karnezis IA, Panagiotopoulos E, Tyllianakis M, Megas P, Lambiris E (2005). Correlation between radiological parameters and patient-rated wrist dysfunction following fractures of the distal radius. Injury.

[26] Chou YC, Chen AC, Chen CY, Hsu YH, Wu CC (2011). Dorsal and volar 2.4-mm titanium locking plate fixation for AO type C3 dorsally comminuted distal radius fractures. J Hand Surg.

